# Expression of Aquaporin 5 (AQP5) Promotes Tumor Invasion in Human Non Small Cell Lung Cancer

**DOI:** 10.1371/journal.pone.0002162

**Published:** 2008-05-14

**Authors:** Young Kwang Chae, Janghee Woo, Mi-Jung Kim, Sung Koo Kang, Myoung Sook Kim, Juna Lee, Seung Koo Lee, Gyungyub Gong, Yong Hee Kim, Jean Charles Soria, Se Jin Jang, David Sidransky, Chulso Moon

**Affiliations:** 1 Department of Otolaryngology – Head and Neck Surgery, Asan Medical Center, College of Medicine, Ulsan University, Seoul, Republic of Korea; 2 Department of Pathology, Asan Medical Center, College of Medicine, Ulsan University, Seoul, Republic of Korea; 3 Graduate Program in Human Genetics, Johns Hopkins University, Baltimore, Maryland, United States of America; 4 Department of Oncology, Johns Hopkins University, Baltimore, Maryland, United States of America; 5 Department of Medicine, Gustave-Roussy Institute, Villejuif, France; City of Hope Medical Center, United States of America

## Abstract

The aquaporins (AQP) are water channel proteins playing a major role in transcellular and transepithelial water movement. Recently, the role of AQPs in human carcinogenesis has become an area of great interest. Here, by immunohistochemistry (IHC), we have found an expression of AQP5 protein in 35.3% (IHC-score: ≥1, 144/408) of the resected NSCLC tissue samples. Cases with AQP5-positive status (IHC-score: ≥2) displayed a higher rate of tumor recurrence than negative ones in NSCLC (54.7% vs. 35.1%, p = 0.005) and worse disease-free survival (p = 0.033; OR = 1.52; 95%CI:1.04−2.23). Further *in vitro* invasion assay using BEAS-2B and NIH3T3 cells stably transfected with overexpression constructs for full length wild-type AQP5 (AQP5) and its two mutants, N185D which blocks membrane trafficking and S156A which blocks phosphorylation on Ser156, showed that AQP5 induced cell invasions while both mutants did not. In BEAS-2B cells, the expression of AQP5 caused a spindle-like and fibroblastic morphologic change and losses of cell-cell contacts and cell polarity. Only cells with AQP5, not either of two mutants, exhibited a loss of epithelial cell markers and a gain of mesenchymal cell markers. In a human SH3-domains protein array, cellular extracts from BEAS-2B with AQP5 showed a robust binding activity to SH3-domains of the c-Src, Lyn, and Grap2 C-terminal. Furthermore, in immunoprecipitation assay, activated c-Src, phosphorylated on Tyr416, showed a stronger binding activity to cellular extracts from BEAS-2B with AQP5 compared with N185D or S156A mutant. Fluorescence in situ hybridization (FISH) analysis failed to show evidence of genomic amplification, suggesting AQP5 expression as a secondary event. Based on these clinical and molecular observations, we conclude that AQP5, through its phosphorylation on Ser156 and subsequent interaction with c-Src, plays an important role in NSCLC invasion and, therefore, may provide a unique opportunity for developing a novel therapeutic target as well as a prognostic marker in NSCLC.

## Introduction

The aquaporins (AQP) represent a family of transmembrane water channel proteins widely distributed in various tissues throughout the body and play a major role in transcellular and transepithelial water movement [1], [2]. So far, at least ten distinct AQPs have been characterized in humans and there has been an increasing understanding of their roles in human pathophysiology [2]. However, only recently has data emerged on the role of human AQPs (hAQPs) as one of the key elements directly involved in human carcinogenesis [3]. Expression of hAQP1 is frequently related with colon cancer, pancreatic cancer, brain tumor, renal cell carcinoma, and microvessels of (MM), paralleling angiogenesis [4]–[8]. Likewise, expression of AQP5 was increased in pancreatic cancer and ovarian cancer [7], [9]. Moreover, we have previously reported the induction of AQP5 expression in its message during the early colon cancer development [5].

At the functional level, AQP1 is shown to be one of the delayed early response genes and also involved in cell migration, and angiogenesis [10], [11], and expression of AQP1, AQP3 and AQP5 were induced during lymphocyte activation [12]. Previously, we have provided evidence for novel oncogenic properties of AQP1 and its expression in resected tissue samples from non small cell lung cancer. Further evidence of the role of AQP5 in human carcinogenesis was also provided by our group [13], [14]. Ectopic expression of AQP5 in NIH3T3 cells induced many phenotypic changes characteristic of transformation both *in vitro* and *in vivo* by promoting signaling pathways activated through Ras, which is induced by phosphorylation of the PKA consensus site of AQP5.

In this study, we investigated the role of AQP5 in lung cancer. AQP5 was chosen based on a number of studies: First, our preliminary study showed that, among AQP1, 3, and 5, AQP5 showed the most robust oncogenic potential in NIH3T3 cell line. Second, although both AQP1 and AQP5 showed oncogenic property with NIH3T3 cell line, AQP5 expression induced ERK activation [13], while AQP1 expression did not [15]. Third, the expression of AQP5, not AQP1, or AQP3, was found to be associated with Ras/ERK/Rb pathway activation in colon cancer cell lines and was linked with liver metastasis in colon cancer patients [16]. We have previously demonstrated the expression of AQP1 in human primary lung cancer tissues; 18 out of 44 samples of non small cell lung cancer patients showed positive AQP1 protein expression. However, it has been shown that AQP5 showed more robust oncogenic potential than AQP1 as well as AQP3 in soft agar assay, focus formation assay, and cell proliferation (MTT) assay [13]–[15], leading us to choose AQP5 for its role in lung carcinogenesis not only for clinical validation study but also for studies in its underlying molecular mechanisms.

Here, we present both molecular and clinical evidence that AQP5 may play a role in the progression of non small cell lung cancer (NSCLC). First, based on immunohistochemical analysis of hAQP5 with 408 NSCLC tissues, we have investigated whether expression profile of AQP5 in human lung cancer correlates with disease progression and survival. Then, we have performed invasion assay and epithelial-mesenchymal transition marker study in human bronchial epithelial cells overexpressing AQP5 versus its mutants in addition to mock control, followed by further molecular interaction studies to elucidate AQP5 mediated pathway. Moreover, FISH analysis was done to identify molecular mechanisms underlying hAQP5 expression.

## Methods

### Cell culture

All cell lines were obtained from the American Type Tissue Collection (Rockville, MD). The murine fibroblast cell line NIH3T3 was cultured in DMEM in the presence of 10% FBS. The normal lung cell line BEAS-2B was grown in LHC-9 medium in the presence of 0.5 ng/ml recombinant epidermal growth factor (EGF), 500 ng/ml hydrocortisone, 0.005 mg/ml insulin, 0.035 mg/ml bovine pituitary extract, 500 nM ethanolamine, 500 nM phosphoethanolamine, 0.01 mg/ml transferrin, 6.5 ng/ml 3,3′,5-triiodothyronine, 500 ng/ml epinephrine, 0.1 ng/ml retinoic acid (Clonetics Corporation, Walkersville, MD). All cells were cultured in 5% CO_2_ balanced air at 37°C.

### Plasmid constructs and production of stable cell lines

Human cDNA from HEK 293 was amplified by polymerase-chain reaction (PCR) using primers for wild-type AQP5 (AQP5) and then inserted into EcoR I and XhoI site of pcDNA 3.1(+). Clones were confirmed by restriction analysis and by DNA sequencing of both strands. Serine 156 or Asparagine 185 was replaced with alanine or aspartic acid, respectively, by PCR-based site-directed mutagenesis to produce the S156A or N185D AQP5 mutant based on the AQP5 pcDNA3.1 construct as previously described [13]. AQP5 WT and two mutants were inserted into the EcoR I and BamH I sites of p3XFLAG-CMV-14 (SIGMA) and verified by DNA sequencing of both strands of the mutants. Expression constructs were transfected into NIH3T3 and BEAS2B cells with FuGENE 6 according to manufacturer's recommendations. All transfectants were selected with 800 µg/mL G418 for 3 weeks and selected clones were screened with Western blot using AQP5 antibody and/or RT-PCR. When confluent, cells were subcultured by trypsinization (0.05% trypsin, 0.53 mM EDTA in Hanks' balanced salt solution). The pictures that show the morphologies of various stable cells expressing different constructs were taken through phase contrast microscopy (ECLIPSE TE300, Nikon Co., Tokyo, Japan).

### Invasion assay

Matrigel invasion assay was performed with NIH3T3 and BEAS2B cells. BioCoat Matrigel (BD Biosciences, Bedford, MA) that reconstitutes the basal membrane was purchased to assess cell invasion. 24-well tissue culture plate inserts coated with Matrigel were re-hydrated for 2 hours in 37°C media. Media (0.6 ml) containing 5% fetal bovine serum was added to each plate well as a chemoattractant, and 0.2 ml (2×10^4^ cells) of cell suspension was added to each insert. After incubation for 24 hours, non-invading cells were removed from the upper side of the membrane by scrubbing. To minimize the effect of cell proliferation on the invasion assay, we have confirmed that, in the first 24 hours, BEAS-2B cells undergo minimal cell division and do not show any noticeable difference in cell proliferation between cells expressing AQP5 and cells expressing mock vector (Fig. S1). Invasion of cells to the underside of the Matrigel-coated membrane was detected by staining the cells with Mayer's hematoxylin solution and visualizing the cells under a microscope. After staining, cells were counted under a microscope in four randomly selected fields (magnification ×100) and results were expressed in the form of a bar graph. Assays were performed with triplicate wells for each condition.

### Immunoblotting and immunoprecipitation

Lysates from cultured cells and tissues were prepared in ice-cold NP-40 lysis buffer (10 mM Tris-HCl (pH 7.4), 137 mM NaCl, 10% glycerol and 0.1% Nonidet P-40) containing an inhibitor cocktail of 10 mM β-glycerol phosphate, 1 mM phenylmethylsulfonyl fluoride, 10 mM NaF, 10 mM Na orthovanadate, 4.5 U/ml aprotinin (Sigma), and 1 µg/ml leupeptin (Sigma). Crude protein lysates (30 µg) were separated by 12% SDS-PAGE (BIORAD), transferred to nitrocellulose membranes (BIORAD), and blocked for 1 hour with 5% nonfat dry milk in Tris-buffered saline with 0.05% Tween 20. The following commercial antibodies were used for Western blot analysis: anti-AQP5 (1∶200, Alpha Diagnostic), anti-α-catenin antibody (1∶1000, Cell Signaling) anti-γ-catenin antibody (1∶1000, Cell Signaling), anti-fibronectin antibody (1∶1000, Cell Signaling), anti-vimentin antibody (1∶1000, Cell Signaling), anti-E-cadherin antibody (1∶1000, Cell Signaling), anti-c-Src antibody (1∶1000, Cell Signaling), anti-phospho Src antibody (1∶1000, Cell Signaling), anti-Lyn antibody (1∶1000, Cell Signaling), anti-grap2 antibody (1∶1000, Cell Signaling), and anti-β actin antibody (1∶5000; Sigma). Phospho-Src antibody was first used; blots then were stripped and re-blotted with anti-src and beta-actin antibodies. Appropriate anti-rabbit and anti-mouse horseradish peroxidase-conjugated secondary antibodies (1∶3000; Amersham) were used. Immunoreactive bands were detected by enhanced chemiluminescence (Pierce).

### SH3 domain binding

TranSignal SH3 domain array III spotted with peptides representing 34 different human SH3 domains including Grb2 and Src, were purchased from Panomics and tested for their binding to AQP5 segments and AQP5 WT maintaining natural topology according to the manufacturer's instruction. The SH3 domain-containing proteins on the array were spotted in duplicate. Briefly, membrane filters incubated with 30 µg/ml protein for overnight. After washing, protein binding was visualized by incubation with HRP-conjugated His antibody or FLAG antibody. This assay was performed at least twice with each protein.

### Protein identification and interaction

For pull-down assays, GST fusion proteins (SH3 domain-containing proteins) and GST were purchased from Panomics. Cells were lysed in NP-40 lysis buffer, and lysates were then incubated with either GST or GST fusion protein at 4°C for 2 hours, followed by the addition of 20 µl glutathione-Sepharose 4B beads. After 30 minutes of mixing, the beads were washed four times. Proteins were eluted in Laemmli sample buffer and were analyzed by SDS-PAGE followed by immunoblot with indicated antibodies.

### Cancer tissue microarray

Tissue microarrays (TMAs) were constructed using formalin-fixed, paraffin-embedded tissues of 419 NSCLC obtained from the Department of Pathology of Asan Medical Center under the approval of the Internal Review Board (IRB number: 2006-0019). The lung samples were obtained from 1996 to 1999. The original H&E stained slides were reviewed by study pathologists and representative areas of each tumor were arrayed to the triplicated blocks to minimize the tissue loss and overcome the heterogeneity of the tumors.

### Immunohistochemistry

The immunostaining procedures were performed using the Benchmark automatic immunostaining device (Ventana Medical System, Tucson, AZ, USA) with affinity-purified goat antibody raised against the 19-amino acid sequence (aa 251–269) of the COOH-terminus of human AQP5 (Alpha Diagnostic, San Antonio, Texas) at a 1∶50 dilution. Tissue array sections (4 µm-thick) were deparaffinized in xylene, rehydrated in graded alcohols, and treated with 3% hydrogen peroxide in methanol at room temperature for blocking of endogenous peroxidase activity. The AQP5 antibody was visualized using the avidin-biotin-peroxidase technique (DAKO LSAB kit; DAKO Cytomation, Carpinteria, CA) and followed by chromogen detection with diaminobenzidine (DAB). Negative controls were performed by omitting the primary antibody incubation step.

### Scoring of immunohistochemistry

Immunostaining on the TMAs was graded semi-quantitatively considering both staining intensity and percentage of positive tumor cells by two study pathologists (S.J.J. and M.J.K.) blinded to the clinicopathologic variables. The staining intensity was scored on a scale of four grades: 0, no staining of cancer cells; 1, weak staining; 2 moderate staining; 3, strong staining. The percentage of stained tumor cells was graded on a scale of 2 grades: 0, <10% and 1, >10%. AQP5 expression in the cancer tissue was defined as positive when the product of intensity score by percentage score is 1 or more. Both histological type and grade were confirmed on hematoxylin and eosin (H&E) stained TMA slides.

### Fluorescence in situ hybridization analysis

Sixty-nine NSCLCs which had an expression of AQP5 (score = 2 or 3) were reassembled in two TMA blocks for FISH analysis, each. Sections (5 µm thick) of the TMA slides were deparaffinized and pretreated for FISH. The probe for AQP5 detection was derived from Homo sapiens 12 BAC RP11-469H8 containing the whole AQP5 gene (GenBank Accession no. AC025154), and labeled with rhodamin (Macrogen, Seoul, Korea). Two genomic DNA clones with 91 kb and 68 kb sizes franking 12p13.31 region labeled with FITC were used as control probes (Macrogen, Seoul, Korea). FISH was performed using Vysis reagents according to the manufacturer's protocols (Vysis, IL, USA). Slides were counterstained with 4, 6-diamidino 2-phenylindole for microscopy. The signal was counted under ×1000 magnification.

### Statistical Analysis

Statistical analyses were performed using the chi-square test with SPSS software (SPSS 12.0K Inc., Chicago, Illinois, USA). The patients' mean age and tumor size were evaluated by t-test. Survival curves were produced using the Cox proportional hazards model to test the risk of cancer death. Prognostic factors were examined by both univariate and multivariate analyses. In multivariate analysis, age, sex, histological grade, and tumor stage were accounted for. We did not control for treatment methods since a majority of the patients underwent surgery and the TNM stage based on which treatment modalities are decided, demonstrated no association with AQP5 expression. All p-values were two-sided, and differences at p<0.05 were considered statistically significant.

## Results

### AQP5 overexpression is associated with worse prognosis in NSCLC

**Figure 1:**
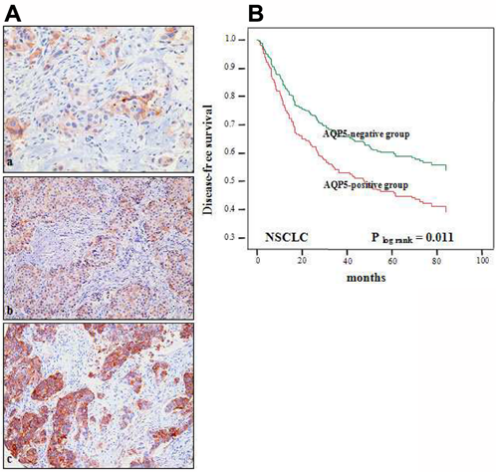
Clinical importance of AQP5 in non small cell lung cancer. (A) Immunohistochemistry of AQP5. Photomicrographs of AQP5 immunostaining in arrayed non small cell lung cancer (NSCLC) tissues. Examples of weak (+1), moderate (+2), and strong (+3) immunoreactivity in NSCLCs (a to c, respectively) Original magnification ×200. (B) Kaplan Meier Curve in NSCLC patients. AQP5-positive cases displayed a less favorable disease-free survival rate (log rank p = 0.011) than AQP5-negative cases.

A significant expression of AQP5 protein was observed in 16.9% (69/408) of NSCLC TMA samples (immunostaining score of 2+; defined as positive) (Table 1, Fig 1A). As shown in the figure 1A, only cancer cells, not tumor-infiltrating lymphocytes, showed AQP5 expression. Including cases with weak expression (immnunostaining score of 1+), it was as high as 35.3% (144/408, Table 1). Interestingly, AQP5 overexpression was observed predominantly in lung adenocarcinomas (p<0.001, Table 2). However, no other statistically significant correlation was found in NSCLCs between AQP5 expression and demographic and pathologic factors, such as age, sex, histological differentiation, and tumor stage (Table 2). Due to the insufficient sample size of metastatic cases (n = 4), no further statistical correlation study on metastasis was performed.

**Table 1:** The immunostaining scores of AQP5 and the histological subtype of NSCLC.

AQP5 immunostaining score	0	1	2	3	Total
**Adenocarcinoma**	101 (54%)	34 (18%)	25 (13%)	28 (15%)	188 (46%)
**Squamous cell carcinoma**	148 (74%)	39 (20%)	6 (3%)	7 (3.5%)	200 (49%)
**Large cell carcinoma**	13 (86%)	0 (0%)	1 (7%)	1 (7%)	15 (4%)
**Otherŝ**	2(40%)	2 (40%)	1(20%)	0 (0%)	5 (1%)
**Total**	264 (65%)	75 (18%)	33 (8%)	36 (9%)	408 (100%)

ˆ Other subtypes include three adenosquamous, one mixed squamous and neuroendocrine carcinoma and one sarcomatoid carcinoma.

**Table 2:** Association between AQP5 overexpression and the demographics of patients and tumor characteristics.

Clinical findings	Lung cancer (AQP(+)/n = 69/408)	p-value*
**Mean Age** (range, yrs)	63.3 (29–86)	0.877
**Gender**		
Woman	34/87 (39.1%)	0.747
Man	93/321 (29.0%)	
**Histological subtype**		
Adenocarcinoma	53/188 (28.2%)	<0.001
Squamous Cell Carcinoma	13/200 (6.5%)	
Large Cell Carcinoma	2/15 (6.7%)	
Others†	1/5 (20.0%)	
**Grade** §		
WD (HG 1)	20/110 (18.2%)	0.571
MD (HG 2)	36/201 (17.9%)	
PD (HG 3)	13/97 (13.4%)	
**Mean tumor size** (cm)	4.2	0.281
**T stage**		
T1	16/74 (21.6%)	0.221
T2	41/276 (14.9%)	
T3	6/35 (17.1%)	
T4	6/20 (30.0%)	
Unknown	0/3 (0%)	
**N stage**		
N0	34/205 (16.6%)	0.196
N1	10/85 (11.8%)	
N2	25/117 (21.4%)	
N3	not available	
Unknown	0/1	
**TNM stage**		
I	31/187 (16.6%)	0.353
II	10/85 (11.8%)	
III	27/129 (20.9%)	
IV	1/4 (25.0%)	
Unknown	0/3 (0%)	

ˆ In this table, AQP5(+) group is defined as cases with moderate and strong expression (score 2, and 3). If all of three groups (groups with expression score of 1, 2, and 3) including the group with weak expression (score 1) are counted, percentage of expression will be significantly high (45.5%, 185/408).

* Pearson *X* ^2^ test was used to derive p values, except for age and tumor size where student t-test was performed.

† Other subtypes include three adenosquamous, one mixed squamous and neuroendocrine carcinoma and one sarcomatoid carcinoma.

§ Lung cancers are graded by the modified Broder's grading system (HG, histological grade; WD, well differentiated; MD, moderately differentiated; PD, poorly differentiated).

To our surprise, cases with AQP5-positive status displayed a higher rate of tumor recurrence than negative ones in NSCLC (54.7% vs. 35.1%, p = 0.005). Although AQP5 overexpression was not statistically significantly related with overall survival, remarkably, those with NSCLC showed a worse disease-free survival rate (log rank test, p = 0.011) (Fig. 1B). Even after adjusting for histological grade and tumor stage, AQP5 overexpression in NSCLC was still significantly associated with earlier disease progression (p = 0.033; OR = 1.52; 95% CI: 1.04–2.23). Therefore, AQP5 status seems to be an independent molecular marker associated with worse clinical outcomes.

### Human AQP5 promotes cell invasion

**Figure 2:**
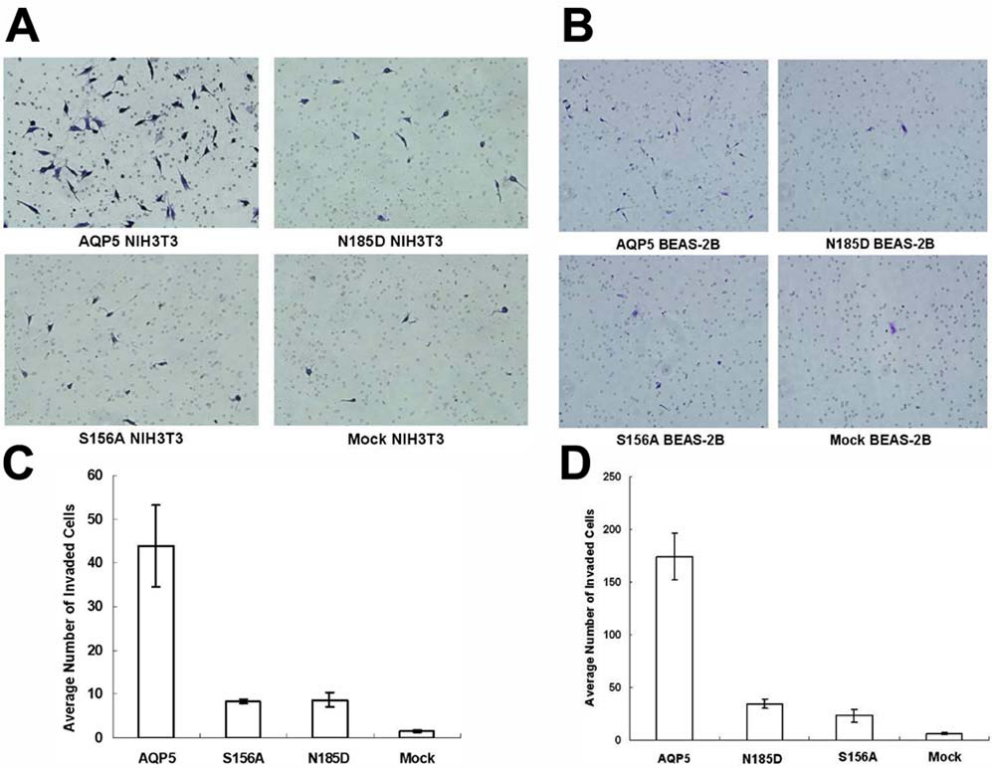
Invasion assay with stable cells expressing AQP5 and mutants. Matrigel invasion assay was performed with each of BEAS-2B cells (A, C) and NIH3T3 cells (B, D) which express AQP5 or each of two mutants. AQP5 induced cell migration and invasion in BEAS-2B, immortalized human bronchial epithelial cells as well as NIH3T3 cells. In contrast, both of the N185D mutant (impaired membrane trafficking) and the S156A mutant (impaired phosphorylation at Ser 156 by PKA) do not induce cell migration and invasion in either BEAS-2B cells or NIH3T3 cells as compared to Mock.

From our clinical findings, we have hypothesized that AQP5 may induce cell invasion, a necessary step for tumor metastasis. In order to investigate whether AQP5 overexpression promotes cell invasion in human bronchial epithelial cells and, at the same time, to determine what components of AQP5 protein plays a role in the process, we first performed Matrigel invasion assay in BEAS-2B cell lines stably expressing AQP5 and its two mutants, N185D which blocks membrane trafficking and S156A which blocks phosphorylation on S156 site of AQP5 molecule [13], [14], [17]. Interestingly, cells with AQP5 demonstrated the highest degree of cell invasion among four groups (Fig. 2A, 2C). Although higher than the mock, the degree of cell invasion in cells with N185D and S156A mutants was clearly less than that of cells with AQP5 (Fig. 2A, 2C). These findings suggest that both membranous expression of AQP5 and its phosphorylation on S156, the PKA consensus site, are important for AQP5 induced cell invasion in BEAS-2B cells. To exclude the possibility that this phenomenon is not unique to human bronchial epithelial cells, we performed the same assay using mouse fibroblast NIH3T3 cell lines, which were transfected with same expression constructs [13] and have observed similar findings (Fig. 2B, 2D).

### AQP5 expression leads to fibroblast-like morphologic change in bronchial epithelial cells

**Figure 3:**
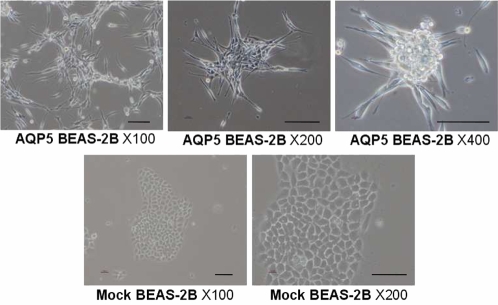
The Morphologies of stable cells expressing AQP5 and mutants. The morphologies of the BEAS-2B human bronchial epithelial cells expressing the mock vector FLAG or FLAG/AQP5 were revealed by phase contrast microscopy. BEAS-2B cells maintained highly organized cell-to-cell adhesion and cell polarity as a typical epithelial morphology. The expression of AQP5 caused a spindle-like and fibroblastic morphology and loss of cell-cell contacts and cell polarity. Scale bars, 50 µm.

We, then, decided to assess any morphologic changes accompanied by AQP5 overexpression. The morphologies of the BEAS-2B human bronchial epithelial cells carrying either the mock vector FLAG or FLAG/AQP5 were examined by phase contrast microscopy. As expected, mock transfected BEAS-2B cells showed highly organized cell-to-cell adhesion and maintained cell polarity, which is the typical morphology of normal epithelial cells (Fig. 3). However, the expression of AQP5 caused a marked spindle-like and fibroblastic change in cell morphology and a loss of cell-to-cell contact and cell polarity (Fig. 3). These morphologic alterations may imply that AQP5 plays a role in epithelial-mesenchymal transition (EMT).

### AQP5 expression is associated with molecular markers of epithelial-mesenchymal transition

**Figure 4:**
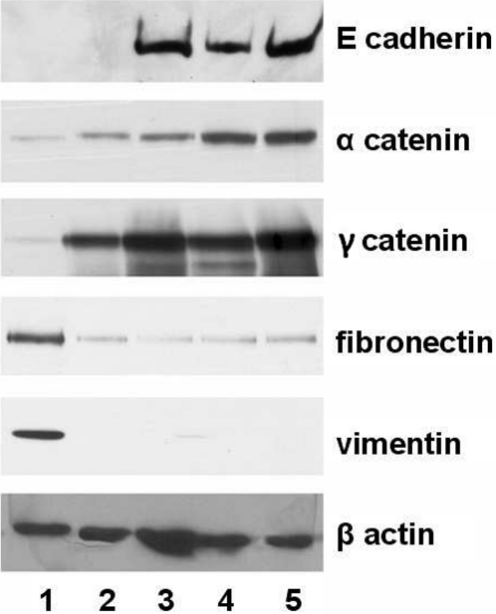
Immunoblotting analysis of epithelial-mesenchymal transition markers in stable cells expressing AQP5 and mutants. Expression of epithelial proteins including E-cadherin, α-catenin and γ-catenin, and mesenchymal proteins including fibronectin and vimentin was examined by immunoblotting in the four separate BEAS-2B cells, each of which carries overexpression construct for AQP5 (clone #1 and #2), N185D, S156A and Mock. Only cells with AQP5 exhibited a loss of epithelial markers and a gain of mesenchymal cell markers. 1, AQP5 clone#1; 2, AQP5 clone#2; 3, N185D; 4, S156A; 5, Mock based on BEAS-2B cell lines.

In order to confirm whether AQP5 really induces EMT in human bronchial epithelial cells, we performed western blot analysis to check protein markers for EMT. Of note, among BEAS-2B cells stably expressing AQP5 (clone #1 and #2), N185D, S156A mutants and mock vector, only cells that express AQP5 exhibited a loss of epithelial cell markers, such as E-cadherin, α catenin and γ catenin, and a gain of mesenchymal cell markers including fibronectin and vimentin (Fig. 4). Both changes in the morphology and the molecular profile of AQP5 overexpressing cells provide firm evidence that AQP5 stimulates bronchial epithelial cells to undergo EMT.

### AQP5 interacts with the c-Src, a potent EMT trigger

**Figure 5:**
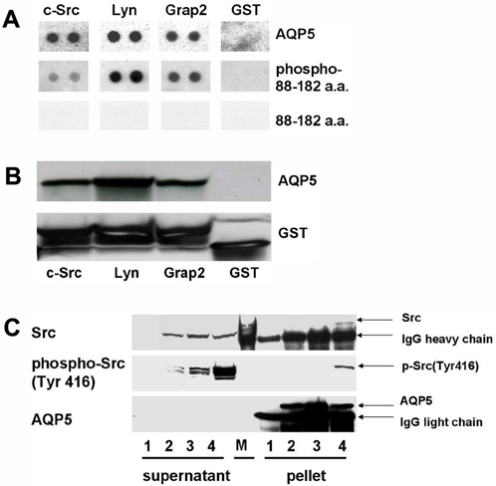
Interaction between AQP5 and the Src family molecules. (A) A human SH3 domains protein array was used to examine the potential binding activities of several SH3 domain-containing proteins to AQP5. Not only AQP5, but also AQP5 segment containing a phosphorylated Loop D (88 aa through 182 aa) were found to bind to the SH3 domain of the c-Src, the Lyn, and the Grap2 protein. The AQP5 segment containing an unphosphorylated Loop D, however, was unable to bind to the SH3 domain of any of these proteins. (B) GST pull-down assay was performed with three different GST fusion proteins: a c-Src SH3 domain, a Lyn SH3 domain, and a C-terminal Grap2 SH3 domain protein. Cells stably expressing AQP5 in human bronchial epithelial cells, BEAS-2B, revealed a strong interaction with the c-Src SH3 domain protein, the Lyn SH3 domain protein and the C-terminal Grap2 SH3 domain protein. (C) AQP5 is co-immunoprecipitated with c-Src in the BEAS-2B cells stably transfected with AQP5 expression construct. Furthermore, only an activated form of c-Src, phosphorylated on Tyr 416, is co-immunoprecipitated with AQP5. 1, Mock; 2, N185D; 3, S156A; 4, AQP5; M, molecular weight marker.

Then, we investigated the possible molecular mechanism behind the EMT promoting property of AQP5. Human AQP5 carries a diproline peptide sequence (RTSPVGSP) in the loop D [18], [19] which bears a sequence similarity to the SH3 binding consensus site. Thus, it is possible that this segment of AQP5 may bind with the SH3 domain of an adaptor molecule [18]. Therefore, we performed a human SH3 domains protein array to examine the potential of several SH3 domain containing proteins to bind to human AQP5. Notably, the purified AQP5 from stable BEAS-2B cells showed binding activity to the SH3 domain of c-Src, Lyn, and Grap2 C-terminal (Fig 5A). The AQP5 segment containing a phosphorylated Loop D (88 aa through 182 aa) was also able to bind the SH3 domain of c-Src, Lyn, and Grap2 C-terminal (Fig. 5A). The AQP5 segment containing an unphosphorylated Loop D, however, was unable to bind to any SH3 domains from three proteins (Fig. 5A). The phosphorylation status of each protein was further confirmed.

In addition, we performed a GST pull-down assay to verify a direct interaction between AQP5 and the Src family molecules. In this assay, we used three different GST fusion proteins: a c-Src SH3 domain, a Lyn SH3 domain, and a C-terminal Grap2 SH3 domain protein, which were identified to interact with AQP5 in SH3 domain protein arrays. Consistent with the result from SH3 domain protein arrays, AQP5 stably expressed in BEAS-2B human bronchial epithelial cells exhibited a strong interaction with the c-Src, Lyn and C-terminal Grap2 SH3 domain proteins (Fig. 5B).

Lastly, to confirm our finding *in vivo*, we performed an immunoprecipitation assay in human bronchial epithelial cell line, BEAS-2B. This time, we focused on c-Src, a molecule well known for its EMT promoting activity [20]. As expected, AQP5 co-immunoprecipitated with c-Src in cells stably expressing AQP5 (Fig. 5C). Intriguingly, c-Src co-immunprecipitated with AQP5 turned out to be an activated form of c-Src, which is phosphorylated at Tyr416 (Fig. 5C). We also note that the cells expressing AQP5 have more activated c-Src than the cells carrying N185D or S156A mutant (Fig. 5C), which suggests that the interaction with AQP5 may be an important step in activating c-Src.

### AQP5 overexpression may not be associated with genomic amplification

To elucidate the molecular mechanisms underlying AQP5 overexpression, we have investigated the presence of genomic amplification through FISH analysis. Out of 69 interpretable cases of NSCLC TMAs, not a single clear pattern of genomic amplification was detected (Fig. S2), which suggests that the ectopic expression of AQP5 could be a secondary molecular event.

## Discussion

We have previously demonstrated that overexpression of AQP5 in mouse fibroblasts, NIH3T3 cells, induces cell proliferation secondary to the activation of Ras, which is mediated by phosphorylation of AQP5 in its PKA consensus site (S156) and this study clearly provided an association between AQP5 and the Ras signal transduction pathway [13], [14]. Additionally, we have recently reported a study which describes an induced expression of AQP5 protein during colorectal carcinogenesis and molecular pathways how AQP5 protein expression can influence colon cancer development by its interaction with the Ras/ERK/Rb signaling pathway [16].

In contrast to these previous reports, here, we have pursued the role of AQP5 on the progression of NSCLC. This is partly based on our prior report, which not only showed the oncogenic property of AQP1 in NIH3T3 cells, but also described the expression profile of AQP1 in NSCLC [15]. This report, although provided certain insight regarding the role of AQPs in NSCLC, did not demonstrate either its clinical implication or related molecular pathways. In our preliminary study for AQP5 in NSCLC, we noticed a series of AQP5 expression in several NSCLC cell lines including H1975, H1838, H1650, H1437, and H838. In addition, similar to our observations with NIH3T3 mouse fibroblast cells [13], [14], we have recently identified that AQP5 overexpression in BEAS-2B cells induces activation of the ERK pathway leading to stimulation of cell proliferation (Kang et al., manuscript in preparation); human bronchial epithelial cells stably transfected with AQP5 demonstrated significantly higher proliferation rate than cells stably transfected with mock (Fig. S1). Based on these preliminary findings and prior observations we had with AQP1 in NSCLC and AQP5 in colorectal cancer study, we have examined the expression profile of AQP5 in a large panel of clinical samples.

While expression of AQP5 in its message level is reported in certain parts of normal human bronchial and alveolar tissues [21], so far, AQP5 expression profiles in NSCLC tissue samples, in its protein level, have not been reported. In order to find clinical implications of AQP5 overexpression in NSCLC, we have gathered resected human NSCLC tissues with an average of five year follow-up, on which we performed immunochemistry to examine the protein expression level of AQP5. While we found AQP5 expression in cancer cells, we did not see any evidence of AQP5 immunoreactivity among tumor-infiltrating lymphocytes, which is contrary to our previous findings showing an induced expression of AQP5 messages during lymphocytes activation [12]. Although this discrepancy can be a result of multiple reasons, it is possible that AQP5 expression during lymphocyte activation can be a temporary event and that, once lymphocytes are activated, they may downregulate AQP5 expression.

We have identified three interesting expression profiles of AQP5 protein in NSCLC with clinical correlation. First, an expression of AQP5 protein is noticed in 35.3% (144/408, Table 1.) of the resected NSCLC tissue samples. Second, NSCLC cases with AQP5-positive status displayed a higher rate of tumor recurrence than negative ones (54.7% vs. 35.1%, p = 0.005). Third, AQP5 overexpression in NSCLC was significantly associated with earlier disease progression (p = 0.033; OR = 1.52; 95% CI 1.04−2.23) and worse disease-free survival. We believe that this is the first clinical evidence indicating a strong correlation between AQP5 expression and the outcome of cancer patients who underwent surgery, and further suggest that hAQP5 is a potential independent prognostic marker. Moreover, the fact that this study is based on a substantial size of patient samples (more than four hundred cases) with an average of 5 year follow-up corroborates our findings.

The prognostic marker for lung cancer is crucial as there are still on-going controversies regarding the post-operative management and follow-up plans for NSLCLC. Although the needs and the effects of post-operative chemotherapy are relatively well established, there seem to be few candidate prognostic biomarkers from which the decisions for chemotherapy can be made [22], [23]. Part of this problem stems from the fact that most of the studies were based on the sample size of less than 200 with an average follow-up year of less than 5 years [23]. Only a few molecular marker studies published used a sample size of above 400 [24], [25]. Looking at a 50% increase in terms of recurrence risk in AQP5 positive group vs. negative group among more than 400 samples with up to 7 year follow-up, study of AQP5 expression in addition to other prognostic markers in surgical samples may provide a dependable guidance for making a better informed decision for postoperative management in NSCLC patients.

Based on our observations with clinical samples from NSCLC and also from our previous observation in colon cancer [16], where AQP5 expression was detected in patients with liver metastasis, we hypothesized that AQP5 is associated with cell invasion. For our invasion assay, we have decided to use BEAS-2B stable cell lines stably transfected with AQP5 overexpression construct and those of two mutants. BEAS-2B is a SV-40 transformed immortalized cell lines originated from normal lung epithelium that has been used extensively as a model cell line to examine the molecular events leading to NSCLC development [26]. In fact, we had previously reported that in BEAS-2B cells, membranous expression of AQP5 is not necessarily associated cAMP induced phosphorylation of AQP5 [17]. In this study, we have used full length wild-type AQP5(AQP5) and its two mutants, S156A and N185D, to examine the effect of PKA pathway (S156A) and NPA motif related with membrane trafficking (N185D), respectively, on cell invasion activity of BEAS-2B. We did so based on our previous finding that in NIH3T3 mouse fibroblast cells, while decreased cell proliferation in S156A mutant was due to the poor activation of Ras/ERK pathway, in the case of N185D mutant, it was owing to the improper trafficking of AQP5 into the cell membrane [13]. In this study, AQP5 induced cell invasion in BEAS-2B cells as well as NIH3T3 cells. In contrast, both of the N185D and S156A mutants failed to induce any significant cell invasion in either BEAS-2B cells or NIH3T3 cells compared with mock. Overall, our result suggests that both phosphorylation of AQP5 in PKA site (S156A) and membrane targeting (N185D) are likely to be critical for the enhanced cell invasion induced by AQP5.

Of note, we have found that the expression of AQP5 in BEAS-2B cells promotes both cell invasion and proliferation. Interestingly, even before we see the effects of cell proliferation, cells become highly invasive (Fig. 2, Fig S1). This may explain, in part, why some of the lung cancers are highly invasive in nature and easily metastasize even when the size of tumor mass is small.

Additionally, the expression of AQP5 in BEAS-2B cells caused a spindle-like and fibroblastic morphology and a loss of cell-to-cell contacts and cell polarity. We have not found comparable changes in cell morphology with mouse NIH3T3 cell line which is already a fibroblast cell line and various other cancer cell lines including colon cancer cell lines (HCT116, DLD1) and leukemic cell lines (K562, LAMA-84). In our previous findings with AQP1 in lung cancer [15], no notable morphologic changes in AQP1 transfected cells were reported. We surmise that the transfection of AQP5 into the normal epithelial cell line may have played a role in inducing fibroblast-like morphologic alteration. However, further transfection of different types of AQPs into normal human bronchial epithelial cell line is planned.

Expression assay of epithelial proteins such as E-cadherin, α-catenin and γ-catenin, and mesenchymal proteins including fibronectin and vimentin in the BEAS-2B cells transfected with AQP5 and its two mutants demonstrated that only cells with AQP5, but none of the mutants, exhibited a loss of epithelial cell markers and a gain of mesenchymal cell markers. Therefore, both morphological modification and molecular profile change suggest that AQP5 expression in BEAS-2B cells induces EMT, thus promoting cell invasion and metastasis.

To elucidate the molecular interaction between AQP5 and its downstream pathway leading to cell invasion with enhanced EMT activity, we hypothesized that the diproline peptide sequence (RTSPVGSP) in loop D of AQP5 is likely to be involved [18], [19] in its interaction with other adaptor proteins. Due to its sequence similarity to SH3 binding consensus, it is possible that this particular sequence can directly bind with the SH3 domain of certain adaptor molecules [18], [19]. Therefore, to identify candidate adaptor molecules which, directly or indirectly, bind to AQP5 through this particular amino acid sequences, we have screened a human SH3 domains protein array. Interestingly, cellular extracts from AQP5 showed a robust binding to SH3 domains of several key proteins associated with signal transduction including c-Src, Lyn, and Grap2 (Fig. 5A). Particularly, a phosphorylated Loop D fragment of AQP5 (88 aa through 182 aa) was able to bind to the SH3 domains of c-Src, Lyn, and Grap2 C-terminal as strongly as AQP5, while unphosphorylated Loop D fragment was not (Fig. 5A). Of note, our competitive inhibition assay using phosphorylated and unphosphoryalated Loop D fragment showed that only the phosphorylated Loop D fragment inhibited cell proliferation in NIH3T3 cells (Lee et al., manuscript submitted), further suggesting that phosphorylation of Loop D is likely to be critical in the interaction of AQP5 with its adaptor molecules involved in the signal transduction pathways of cell proliferation, migration, and invasion such as c-Src [27]. These findings altogether indicate that both the diproline peptide sequence in Loop D and phosphorylation of Ser156 inside the diproline peptide sequence by PKA are required to interact with Src family molecules.

Src is a non-receptor cytoplasmic tyrosine kinase associated with invasive and metastatic phenotype in various tumors and has been known to promote EMT [27]–[29]. The role of c-Src has also been emphasized in the carcinogenesis of NSCLC [30]. The activated, or the phosphorylated form (Tyr416) of c-Src favors invasion by disrupting E-cadherin dependent cell-to-cell contact and enhancing integrin mediated cell-matrix adhesion [31]. Our finding with c-Src is important because AQP5 seems to interact only with the activated form of c-Src (Fig. 5C). More interestingly, the cells with AQP5 were shown to have more activated c-Src than the cells with N185D or S156A mutant (Fig. 5C), hinting that the interaction with AQP5 may induce a conformational change in the inactivated c-Src, enabling it to become phosphorylated, and thus, activated. Overall, our findings on the interaction of AQP5 with c-Src, based on several experiments: *in vitro* SH3 domain protein array and GST pull-down assay and *in vivo* immunoprecipitation, offers a plausible explanation for the molecular mechanism of AQP5 mediated enhanced invasion and EMT.

Finally, to determine the molecular mechanisms underlying the expression of AQP5 in NSCLC, we performed FISH analysis. We were unable to detect any clear evidence of genomic amplification, which indicates that AQP5 expression is likely to be a secondary event. Of note, this finding is similar to the case for AQP1, where no evidence for genomic amplification was identified [15]. Further efforts to identify promoter element responsible for the induced expression of AQP5 in several tumor cell lines suggested several potential cis-acting elements responsible for promoter activity. In addition, methylation analysis of AQP5 promoter regions suggested a possible role of promoter demethylation as a mechanism of AQP5 expression in NSCLC (Kim et al., unpublished result). However, these results are still preliminary and warrant further validation in the future.

We have previously reported that the phosphorylation of AQP5 at the PKA consensus site may play a role in promoting cell transformation [13]. In a more recent study, phosphorylation of AQP5 was reported in the tissues of human non small cell lung cancer, but not in normal lung tissues [14]. While it is possible that activation of AQP5 may lead to increase in its water permeability and thus increase the metabolic capacity of the cell, it appears to be unlikely, as data in Xenopus oocytes suggest otherwise [32]. Yang et al. reported that water channel (including AQP5) phosphorylation by protein kinases A and C did not affect water permeability [32]. Although, there still exists a possibility that AQP5 mediated water transport may, in part, play a role in cell migration as in AQP4 [33], the role of water transport may not be significant compared with the suggested c-Src mediated epithelial mesenchymal transition (EMT) promoting property of AQP5.

In summary, our clinical and molecular findings support our hypothesis that AQP5 may play a critical role in the progression of NSCLC through enhanced cell invasion. This contrasts to other reports showing the role of AQPs in cell proliferation (AQP1 and AQP5) or angiogenesis (AQP1) [11], [13]–[15]. It, further, makes hAQP5 an attractive molecular marker for predicting cancer prognosis. Moreover, a significant expression level of AQP5 (35.3%, 144/408) in NSCLC and its association with worse clinical outcomes may warrant a future study to explore AQP5 as a novel therapeutic target for the management of NSCLC. For example, one of the standard second line therapies of lung adenocarcinoma in the U.S. is the use of epidermal growth factor (EGFR) antagonist, erlotinib, which inhibits tyrosine kinase activity of EGFR [34]. We surmise that in the future, AQP5 antagonists, possibly through inhibition of phosphorylation in S156 or its interaction with downstream pathways, may be used for the management of NSCLC.

## Supporting Information

Figure S1 Cell proliferation assay between BEAS 2B cells transfected AQP5 and mock. (left) Immunoblotting was done to confirm the overexpression of AQP5 in human bronchial epithelial cells stably transfected with AQP5. Mock transfected cells showed no expression of AQP5. Actin was loaded as control. (right) Cell proliferation was measured with MTT assay 1 day and 2 days after seeding at a density of 1×104 cells/well in six-well plates (triplets). After 2 days, cells transfected demonstrated significantly higher proliferation rate than cells transfected with mock (p<0.01).
(0.09 MB DOC)

Figure S2 FISH analysis of AQP5. In the FISH analysis, no genomic amplification is identified in any of 60 NSCLCs tested, and one representative example is shown. (green color: FITC labeled control probe, red color: rhodamin labeled AQP5 probe). Original magnification ×1000 (f).
(0.08 MB DOC)
