# Correction: Expression of Aquaporin 5 (AQP5) Promotes Tumor Invasion in Human Non Small Cell Lung Cancer

**DOI:** 10.1371/annotation/9ae0d68c-71ee-46af-b157-07c34a89bc1f

**Published:** 2008-06-17

**Authors:** Young Kwang Chae, Janghee Woo, Mi-Jung Kim, Sung Koo Kang, Myoung Sook Kim, Juna Lee, Seung Koo Lee, Gyungyub Gong, Yong Hee Kim, Jean Charles Soria, Se Jin Jang, David Sidransky, Chulso Moon

Affiliation 1 is incorrect. It should be: Department of Otolaryngology - Head and Neck Surgery, Johns Hopkins University, Baltimore, Maryland, United States of America

