# Supplementary figures and images for: Expression of Aquaporin 5 (AQP5) Promotes Tumor Invasion in Human Non Small Cell Lung Cancer
Source: PLoS One. 2008 May 14;3(5):e2162. doi: 10.1371/journal.pone.0002162 (PMC2364652; doi:10.1371/journal.pone.0002162)

**Figure S1.**


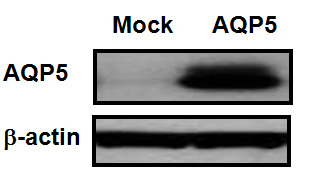
 **
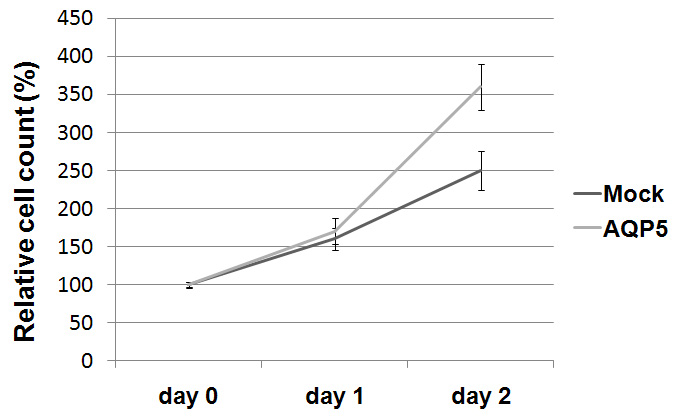
**

Supplement: Figure S1 — Cell proliferation assay between BEAS 2B cells transfected AQP5 and mock. (left) Immunoblotting was done to confirm the overexpression of AQP5 in human bronchial epithelial cells stably transfected with AQP5. Mock transfected cells showed no expression of AQP5. Actin was loaded as control. (right) Cell proliferation was measured with MTT assay 1 day and 2 days after seeding at a density of 1×10^4^ cells/well in six-well plates (triplets). After 2 days, cells transfected demonstrated significantly higher proliferation rate than cells transfected with mock (p<0.01). (0.09 MB DOC) [file pone.0002162.s001.doc]

**Figure S2.**


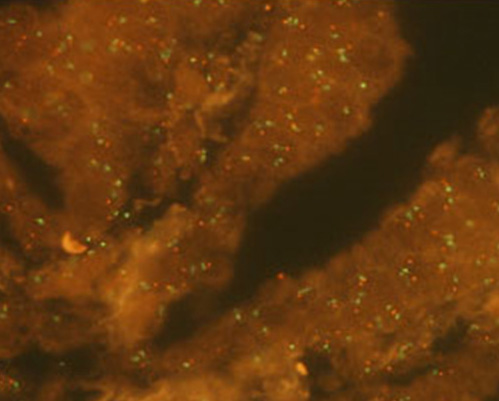

Supplement: Figure S2 — FISH analysis of AQP5. In the FISH analysis, no genomic amplification is identified in any of 60 NSCLCs tested, and one representative example is shown. (green color: FITC labeled control probe, red color: rhodamin labeled AQP5 probe). Original magnification ×1000 (f). (0.08 MB DOC) [file pone.0002162.s002.doc]
